# Exacerbation of substrate toxicity by IPTG in *Escherichia coli* BL21(DE3) carrying a synthetic metabolic pathway

**DOI:** 10.1186/s12934-015-0393-3

**Published:** 2015-12-21

**Authors:** Pavel Dvorak, Lukas Chrast, Pablo I. Nikel, Radek Fedr, Karel Soucek, Miroslava Sedlackova, Radka Chaloupkova, Víctor de Lorenzo, Zbynek Prokop, Jiri Damborsky

**Affiliations:** Loschmidt Laboratories, Department of Experimental Biology and Research Centre for Toxic Compounds in the Environment RECETOX, Faculty of Science, Masaryk University, Kamenice 5/A13, 625 00 Brno, Czech Republic; International Clinical Research Center, St. Anne’s University Hospital, Pekarska 53, 656 91 Brno, Czech Republic; Systems and Synthetic Biology Program, Centro Nacional de Biotecnología CNB-CSIC, Cantoblanco, 28049 Madrid, Spain; Institute of Biophysics, Academy of Sciences of the Czech Republic, v.v.i., Kralovopolska 135, 612 65 Brno, Czech Republic; Department of Histology and Embryology, Faculty of Medicine, Masaryk University, 625 00 Brno, Czech Republic; Department of Experimental Biology, Faculty of Science, Masaryk University, 625 00 Brno, Czech Republic

**Keywords:** Metabolic burden, Substrate toxicity, *Escherichia coli*, Heterologous metabolic pathway, Isopropyl β-D-1-thiogalactopyranoside, Lactose, 1,2,3-trichloropropane, Metabolic engineering

## Abstract

**Background:**

Heterologous expression systems based on promoters inducible with isopropyl-β-D-1-thiogalactopyranoside (IPTG), e.g., *Escherichia coli* BL21(DE3) and cognate LacI^Q^/*P*_*lacUV5*_-T7 vectors, are commonly used for production of recombinant proteins and metabolic pathways. The applicability of such cell factories is limited by the complex physiological burden imposed by overexpression of the exogenous genes during a bioprocess. This burden originates from a combination of stresses that may include competition for the expression machinery, side-reactions due to the activity of the recombinant proteins, or the toxicity of their substrates, products and intermediates. However, the physiological impact of IPTG-induced conditional expression on the recombinant host under such harsh conditions is often overlooked.

**Results:**

The physiological responses to IPTG of the *E. coli* BL21(DE3) strain and three different recombinants carrying a synthetic metabolic pathway for biodegradation of the toxic anthropogenic pollutant 1,2,3-trichloropropane (TCP) were investigated using plating, flow cytometry, and electron microscopy. Collected data revealed unexpected negative synergistic effect of inducer of the expression system and toxic substrate resulting in pronounced physiological stress. Replacing IPTG with the natural sugar effector lactose greatly reduced such stress, demonstrating that the effect was due to the original inducer’s chemical properties.

**Conclusions:**

IPTG is not an innocuous inducer; instead, it exacerbates the toxicity of haloalkane substrate and causes appreciable damage to the *E. coli* BL21(DE3) host, which is already bearing a metabolic burden due to its content of plasmids carrying the genes of the synthetic metabolic pathway. The concentration of IPTG can be effectively tuned to mitigate this negative effect. Importantly, we show that induction with lactose, the natural inducer of *P*_*lac*_, dramatically lightens the burden without reducing the efficiency of the synthetic TCP degradation pathway. This suggests that lactose may be a better inducer than IPTG for the expression of heterologous pathways in *E. coli* BL21(DE3).

**Electronic supplementary material:**

The online version of this article (doi:10.1186/s12934-015-0393-3) contains supplementary material, which is available to authorized users.

## Background

*Escherichia coli* is among the most widely used microbial hosts in both fundamental and applied research [1]. *E. coli* strain BL21(DE3) carries an inducible T7 RNA polymerase-dependent expression system that allows for the simple manipulation and tuning of protein production levels, and it has become a laboratory workhorse [1–4]. This strain carrying commercial pET vectors or their derivatives has been the host of choice in numerous studies on recombinant protein expression [5, 6]. More recently, it has found applications as a cell factory for heterologous expression of entire biochemical pathways in the emerging fields of metabolic engineering and synthetic biology [6–10].

Despite its popularity, the *E. coli* BL21(DE3) and LacI^Q^/*P*_*lacUV5*_-T7 expression system suffers from certain drawbacks that mainly stem from the rapid and strong overexpression of recombinant proteins triggered by exposure to the synthetic inducer IPTG. The negative effects on host fitness associated with redirecting cells’ metabolic capacities to achieve high levels of protein production are known as the *metabolic burden* or *metabolic load* [11]. The burden is often attributed to the overconsumption of metabolic precursors (e.g., amino acids, rRNAs, ATP, and reducing power) to fuel the synthesis of non-essential foreign proteins [12] or the energetically demanding maintenance and replication of plasmid vectors bearing heterologous genes and selection markers [13–15]. Fitness costs associated with the activities of the foreign proteins, which may cross-talk with the host’s extant metabolic network [11] and burdens linked to the components of the expression system, such as the IPTG inducer and its import into the cell, are also frequently discussed [16, 17].

In addition to metabolic burden originating from the expression of foreign pathway components, the microbial cell factories used for biosynthesis of value-added chemicals or biodegradation of polluting compounds may be challenged by the toxicity of the processed substrate or its metabolic intermediates. These issues must be accounted for when considering the evolution of metabolic routes for biodegradation in natural and heterologous hosts [18–22]. Toxicity problems have also been encountered during the engineering of biosynthetic pathways for fatty acids, 1,3-propanediol, amorphadiene, taxadiene, and ethanol in *E. coli* [9, 23–27]. In addition, studies on the consolidated bioprocessing of lignocellulose have highlighted the potential adverse effects of inhibitory molecules in biomass-hydrolysate substrates [28]. Our current understanding of cellular responses to the exogenous and endogenous stressors that may be encountered during bioprocesses is extensive [29]. However, the combined effects of multiple simultaneous stresses on the hosts and their engineered induction systems have not been examined in depth.

**Scheme 1:**
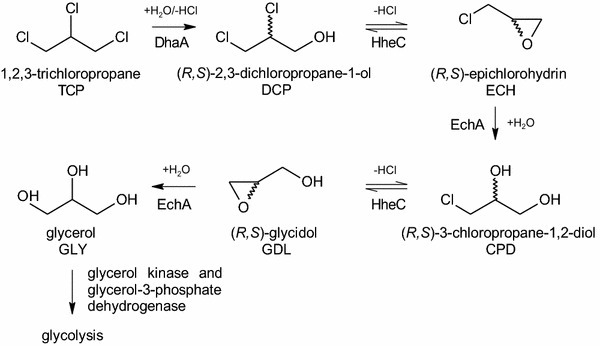
Five-step biotransformation of 1,2,3-trichloropropane into glycerol by the enzymes of the synthetic biodegradation pathway. The pathway consists of haloalkane dehalogenase DhaA from *Rhodococcus rhodochrous* NCIMB 13064 [33] with the haloalcohol dehalogenase HheC and epoxide hydrolase EchA from *Agrobacterium radiobacter* AD1 [34, 35]. Computer-assisted protein engineering was used to improve activity of the haloalkane dehalogenase towards 1,2,3-trichloropropane, leading to the development of the 32-fold more active and 26-fold more efficient mutant DhaA31 [31]. Formed glycerol can be utilized in central catabolic pathways of the host cell

To address this knowledge gap, we examined a recombinant strain of *E. coli* BL21(DE3) under conditions that provide an extreme combination of exogenous and endogenous stresses. The studied strain bears foreign genes encoding a five-step synthetic metabolic pathway for converting the industrial waste product and emerging environmental pollutant 1,2,3-trichloropropane (TCP) into the commodity chemical glycerol (Scheme 1). We have previously assembled this pathway in *E. coli* BL21(DE3) under the control of the LacI^Q^/*P*_*lacUV5*_-T7 system, and used the resulting construct to investigate reported pathway bottlenecks observed in vivo in the environmental bacterium *Agrobacterium radiobacter* AD1 and in vitro in a version of the pathway constructed using immobilized enzymes [20, 22, 30]. By using protein engineering, metabolic engineering, and synthetic biology techniques, we were able to improve the pathway’s performance [22, 31, 32] and identify two important factors limiting its output: an imbalance in the enantioselectivity of the pathway’s enzymes, and the toxicity of the substrate and various pathway intermediates, which reduced the fitness of *E. coli* constructs incubated with TCP [20, 22].

Here, we assess the physiological consequences of using the IPTG-inducible system for the heterologous expression of this TCP degradation pathway. Our results demonstrate a negative synergistic effect of the inducer IPTG and the substrate TCP. Toxicity of TCP in resting *E. coli* BL21(DE3) cells is greatly exacerbated by pre-induction with IPTG. This effect was observed in the cells affected by metabolic burden from corresponding plasmids. Compared to this exacerbation effect, the physiological burden due to the production and presence of the pathway enzymes was minor. Moreover, we show that it is possible to moderate the pathway’s fitness cost by tuning the IPTG concentration or reduce it dramatically by replacing IPTG with the natural inducer lactose. These findings highlight the need to take great care when selecting inducible systems for heterologous expression of metabolic pathways that catalyse harsh reactions, and to finely tune the fitness-productivity trade-off by optimizing the identity and concentration of the inducer.

## Results and discussion

### Biotransformation of TCP by resting *E. coli* BL21(DE3) cells carrying variants of a synthetic degradation pathway

Variants of the synthetic pathway featuring either the wild-type haloalkane dehalogenase or the 26-fold catalytically more efficient mutant DhaA31 were introduced into *E. coli* BL21(DE3) [22]. This host was selected because neither the enzymes nor the metabolites of the TCP pathway occur naturally in its metabolic network and because of the broad repertoire of commercially available Duet vectors for this strain, which enable the tunable co-expression of multiple genes in a single cell [5]. This resulted in the construction of a flexible system with limited risk of metabolic cross-talk, in which the expression of the three pathway components could be manipulated orthogonally [22, 32]. Three previously constructed *E. coli* BL21(DE3) degraders designated degWT, deg31, and deg31opt were tested (Table 1). *E*. *coli* degWT carries a variant of the TCP pathway based on the wild-type DhaA together with HheC and EchA, expressed in a relative ratio of 0.24:0.36:0.40, respectively, as determined after pre-induction with 0.2 mM IPTG (Additional file 1: Fig. S1). Strains deg31 and deg31opt both carry the TCP pathway featuring the engineered dehalogenase DhaA31, but the relative ratio of the three enzymes in deg31 is 0.14:0.41:0.45 while in deg31opt it is 0.63:0.16:0.21. Sodium dodecyl sulfate polyacrylamide gel electrophoresis (SDS-PAGE) experiments indicated that the three pathway enzymes together accounted for a similar proportion of the total soluble protein fraction produced by all three degraders: 52 % for degWT, 54 % for deg31, and 44 % for deg31opt.

**Table 1:** Bacterial strains and plasmids used in this study

Plasmid or strain	Relevant characteristics^a^	Source or reference
Plasmids		
pCDFDuet-1	Expression vector; *CDF* (pCloDF13 replicon), encodes two multiple cloning sites, each of which is preceded by a T7 promoter, a *lac* operator, and a ribosome binding site; Sm^R^	Merck Millipore, Germany
pCDF-dhaAwt	pCDFDuet-1 carrying wild-type *dhaA* gene (encoding haloalkane dehalogenase) from *Rhodococcus rhodochrous* NCIMB 13064	[22]
pCDF-dhaA31	pCDFDuet-1 carrying mutant variant (*dhaA31*) of haloalkane dehalogenase gene	[22]
pETDuet-1	Expression vector; *ColE1* (pBR322 replicon), encodes two multiple cloning sites, each of which is preceded by a T7 promoter, a *lac* operator, and a ribosome binding site; Amp^R^	Merck Millipore, Germany
pETDuet-echA-hheC	pETDuet-1 carrying wild-type *echA* and *hheC* genes (encoding epoxide hydrolase and haloalcohol dehalogenase) from *Agrobacterium radiobacter* AD1	[22]
pACYCDuet-1	Expression vector; *P15A* (pACYC184 replicon), encodes two multiple cloning sites, each of which is preceded by a T7 promoter, a *lac* operator, and a ribosome binding site; Cm^R^	Merck Millipore, Germany
pACYC-echA-hheC	pACYC-1 carrying the wild-type *echA* and *hheC* genes (encoding epoxide hydrolase and haloalcohol dehalogenase) from *Agrobacterium radiobacter* AD1	[22]
*Escherichia coli*		
DH5α	Cloning host; Φ80*lacZ*ΔM15 *recA1 endA1 gyrA96 thi*-*1 hsdR17*(r_K_^−^m_K_^+^) *supE44 relA1 deoR* Δ(*lacZYA*-*argF*)*U169*	Life Technologies, USA
BL21(DE3)	Expression host; F^−^ *ompT gal dcm lon hsdS*_*B*_(r_B_^−^ m_B_^−^) λ(DE3 [*lacI* ^*Q*^ *lacUV5*-*T7 gene 1 ind1 sam7 nin5*])	Life Technologies, USA
Host control	*E. coli* BL21(DE3) transformed with pCDF and pETDuet, control strain with empty plasmids	This study
degWT	*E. coli* BL21(DE3) transformed with pCDF-dhaAwt and pETDuet-echA-hheC	[22]
deg31	*E. coli* BL21(DE3) transformed with pCDF-dhaA31 and pETDuet-echA-hheC	[22]
deg31opt	*E. coli* BL21(DE3) transformed with pCDF-dhaA31 and pACYC-echA-hheC	[22]

^a^Antibiotic markers: *Sm* streptomycin, *Amp* ampicillin, *Cm* chloramphenicol

**Fig. 1:**
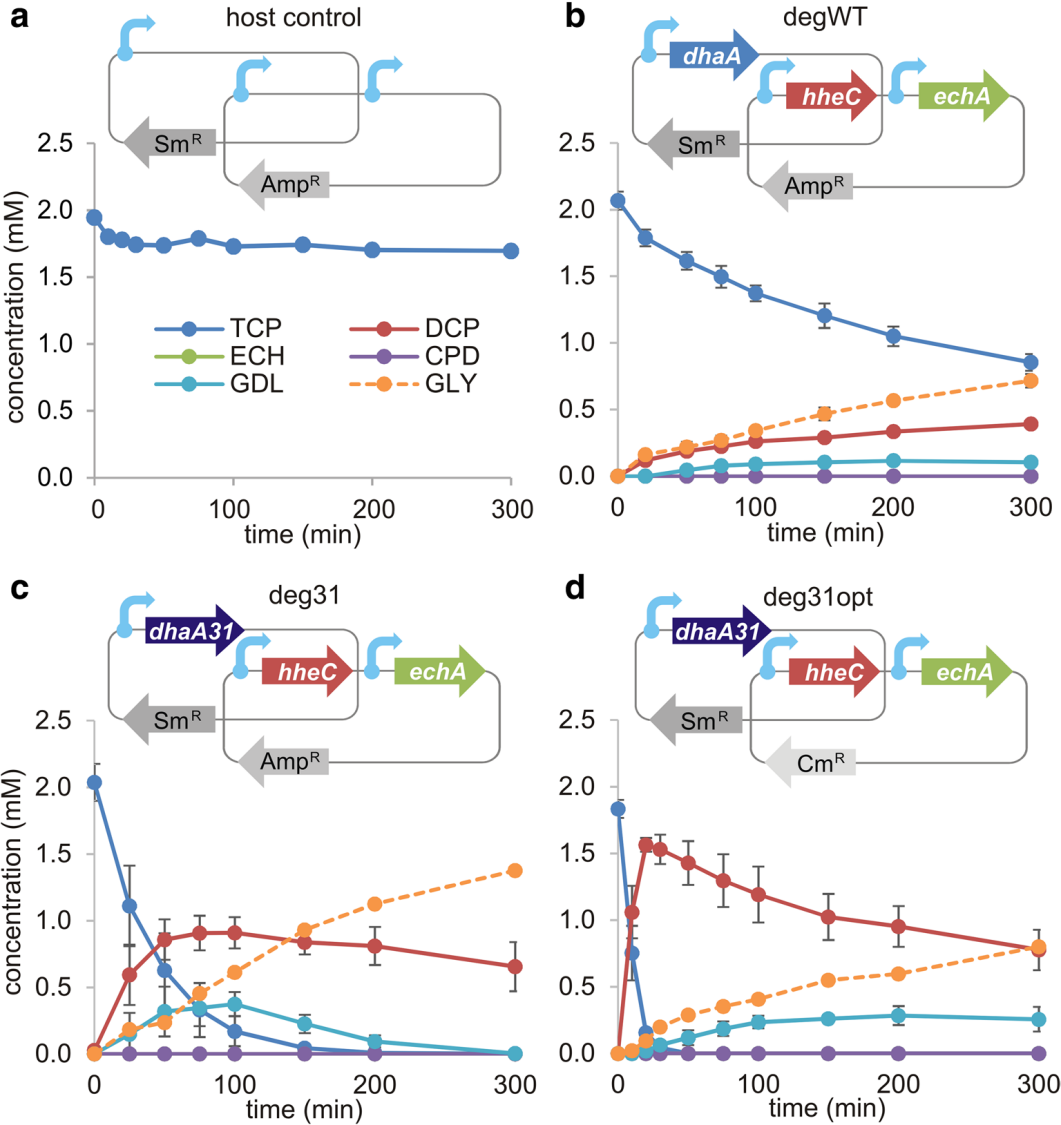
Biotransformation of TCP catalysed by different *Escherichia coli* BL21(DE3) recombinants. **a** Control strain carrying the empty pCDF and pETDuet plasmids with streptomycin and ampicillin resistance marker genes, respectively. The *blue arrows* indicate individual T7 promoters. **b** The degrader degWT, which carries the haloalkane dehalogenase gene (*dhaA*) on pCDF and the haloalcohol dehalogenase (*hheC*) and epoxide hydrolase (*echA*) genes on pETDuet. **c** The degrader deg31, which carries the haloalkane dehalogenase mutant (*dhaA31*) gene on pCDF and two remaining genes of the degradation pathway on pETDuet. **d** The degrader deg31opt, which carries the *dhaA31* gene on pCDF and the two remaining genes of the degradation pathway on pACYC along with a chloramphenicol marker gene. The relative ratios of the TCP pathway genes produced by the degraders degWT, deg31, and deg31opt are 0.24:0.36:0.40, 0.14:0.41:0.45 and 0.63:0.16:0.21, respectively; the corresponding theoretical conversions of TCP into glycerol (GLY) are 35, 68, and 44 %, respectively. *Error bars* represent standard deviations calculated from three independent experiments. Theoretical concentrations of GLY were calculated from experimentally determined concentrations of TCP and intermediates. *Sm* ^*R*^ streptomycin marker gene; *Amp* ^*R*^ ampicillin marker gene; *Cm* ^*R*^ chloramphenicol marker gene; *DCP* 2,3-dichloropropan-1-ol; *ECH* epichlorohydrin; *CPD* 3-chloropropane-1,2-diol; *GDL* glycidol. Note that the *green line* representing ECH is not visible because this intermediate does not accumulate at detectable levels during the experiment

Pre-induced (0.2 mM IPTG) resting cells of each of the recombinant strains and a host control (Table 1) were incubated in phosphate buffer with 2 mM TCP, and the time-courses of TCP biotransformation over a 5 h interval were recorded (Fig. 1). The reaction profiles revealed fundamental differences between the strains with respect to the initial rates of TCP dehalogenation, accumulation of intermediates, and overall efficiency of glycerol formation. The theoretical concentrations of glycerol, otherwise rapidly utilized by *E. coli*, could be calculated from the experimental concentrations of TCP and detected intermediates by virtue of the pathway’s orthogonal nature [22, 32]. While deg31opt benefited from the fastest first step (Fig. 1d), the best balanced pathway with the highest glycerol production was deg31 (Fig. 1c). On the other hand, degWT suffered from slow TCP conversion (Fig. 1b), prolonged exposure to the toxic substrate, and insufficient pathway output. As expected, the host control (carrying the corresponding empty plasmids, Fig. 1a) showed no activity toward TCP in the closed batch system.

The three *E. coli* recombinants and the control strain lacking the synthetic route represent a suitable model system for studying the contribution of metabolic burden and substrate/metabolite toxicity to the fitness cost of TCP biotransformation by whole-cell catalysts.

### Assessment of metabolic burden and substrate toxicity effects by plating

Cell viability, estimated by plating, is a key physiological parameter that should reflect the individual strains’ ability to cope with the stresses caused by the metabolic burden and TCP exposure [36]. *E. coli* degraders pre-induced with 0.2 mM IPTG and host controls were plated before and after 5 h of incubation in phosphate buffer with or without 2 mM TCP. The percentage of surviving cells was calculated after incubation.

**Fig. 2:**
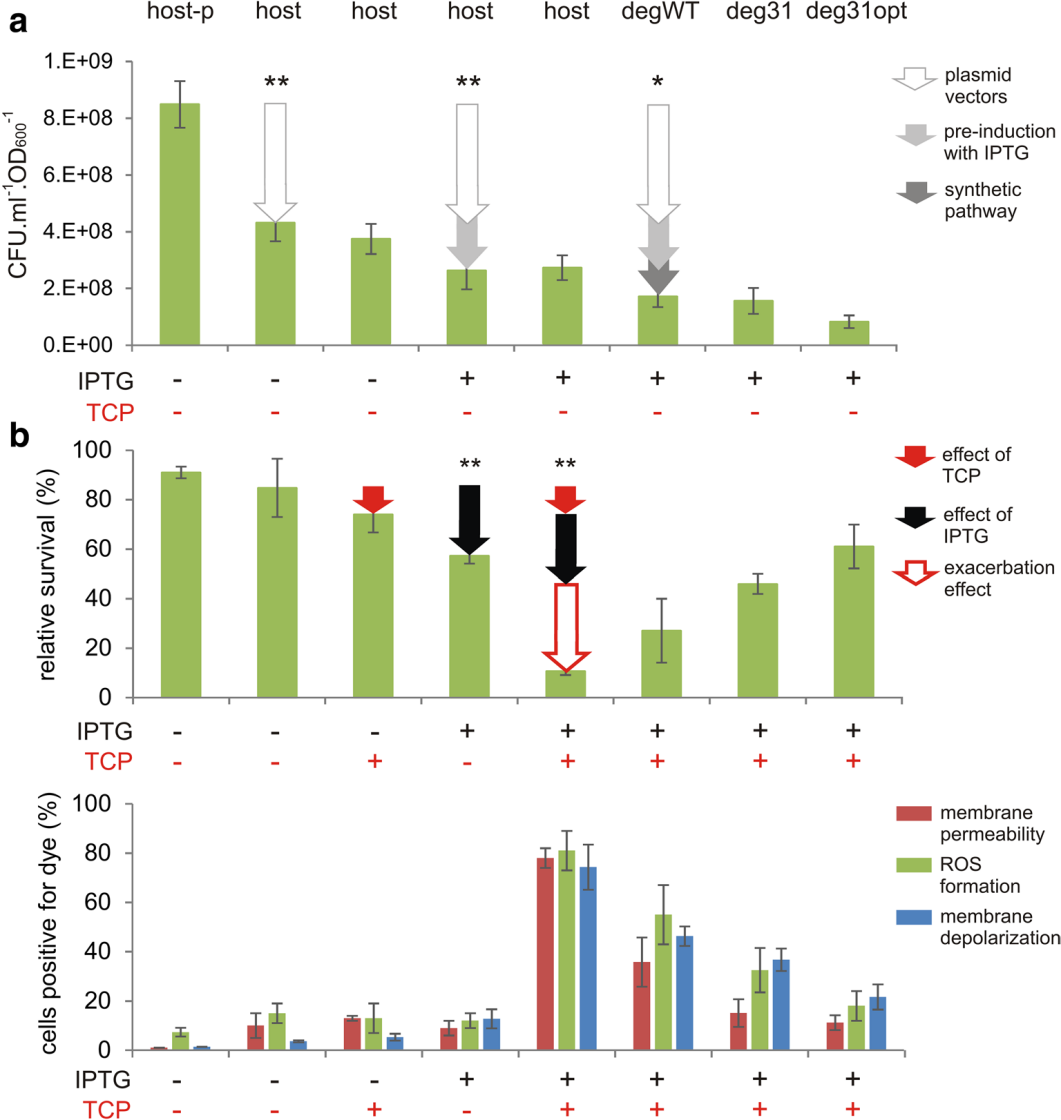
Effects of metabolic burden and TCP toxicity on physiological parameters of *Escherichia coli* BL21(DE3) cells and three recombinants expressing the synthetic metabolic pathway. **a** Viability of cells non-induced or pre-induced with IPTG determined by plating before incubation in phosphate buffer. The effects of metabolic burden stemming from the presence of plasmids, pre-induction with 0.2 mM IPTG, and expression of the synthetic pathway are indicated by *coloured arrows*. *Asterisks* denote significance in decrease of cell count caused by each of three effects at either *P* < 0.05 (*) or *P* < 0.01 (**) when compared with preceding condition. **b** The percentage of surviving cells (*upper graph*) and the corresponding physiological parameters determined by flow cytometry (*lower graph*) after incubation in buffer with or without 2 mM TCP. The separate effects of TCP, IPTG, and the exacerbation of TCP toxicity in cells pre-induced with IPTG are indicated by *coloured arrows*. *Asterisks* denote significant difference in the decrease of cell count caused by each of three effects at *P* < 0.01 when compared with preceding condition. Physiological parameters including membrane permeability, formation of reactive oxygen species (ROS), and membrane depolarization were evaluated by staining the cells with appropriate dyes as explained in the Methods section. *Error bars* represent standard deviations calculated from at least five independent experiments. *CFU* colony forming units; *host-p* *E. coli* BL21(DE3) without plasmids; *host* *E. coli* BL21(DE3) with the empty pETDuet and pCDF plasmids

The data obtained from plating before incubation (Fig. 2a) illustrate the separate effects of individual elements of the overall metabolic burden imposed on the cells. Several factors, including the presence of plasmid DNA and the associated selection markers, the addition of IPTG, and the burden due to heterologous pathway expression affected the degraders’ viability in parallel even before the addition of the toxic substrate. The most pronounced impact at this stage was due to plasmid maintenance and the associated constitutive expression of selection marker genes from the Duet vectors, as well as the presence of IPTG. The presence of two medium-to-high copy plasmids pCDF (20–40 copies per cell) and pETDuet (~40 copies per cell) reduced viability by 50 % (*P* < 0.01; Fig. 2a). The “pre-induction” of the host control with empty plasmids reduced viability by almost 40 % relative to the non-induced control (*P* < 0.01). The expression of pathway enzymes in the *E. coli* degraders further reduced viability by about 20 % (*P* < 0.05). Additional reduction of viability of deg31opt compared to degWT and deg31 can be potentially attributed to the difference in antibiotic selection markers among three recombinants.

The data collected after 5 h incubation with or without TCP (Fig. 2b and Additional file 1: Fig. S2) included several unexpected observations. Surprisingly, TCP (initially added at a concentration of 2 mM) had only minor or negligible effects on the viability of non-induced control cells bearing empty plasmids; there was no significant difference in viability between these cells and the host controls that were exposed to neither TCP nor IPTG. This was unexpected because TCP has been reported to strongly inhibit growing cells of *E. coli* BL21(DE3) and natural hosts such as *A. radiobacter* AD1 or *Pseudomonas putida* MC4 even at concentrations 50 % lower than those used here [20, 22, 37]. On the other hand, the detrimental effect of IPTG was statistically significant (*P* < 0.01) (Fig. 2b and Additional file 1: Fig. S2). The most striking observation was that the relative viability of cells pre-induced with IPTG and then exposed to TCP was almost 90 % lower than that of host controls exposed to neither substance (*P* < 0.01) (Fig. 2b). This dramatic loss of viability does not correspond to a simple sum of the two compounds’ individual effects; it is obvious that the IPTG exacerbated the toxicity of TCP. The fact that IPTG exacerbated the toxicity of TCP and not vice versa was confirmed by plating of three recombinants bearing the synthetic biodegradation pathway (Fig. 2b). Because these degraders had functional pathways for TCP degradation, they were better able to tolerate its presence. Importantly, the faster the conversion of TCP by the pathway enzymes, the greater the degraders’ viability. Deg31opt, which achieved a rapid initial conversion of TCP but accumulated significant quantities of the intermediates DCP and GDL, survived the 5 h incubation almost as well as the host control that was not exposed to the substrate. These data are consistent with the previously reported results of growth arrest tests, which indicated that TCP is the most toxic compound in the pathway [22].

### Assessment of metabolic burden and substrate toxicity effects by multi-parameter flow cytometry

Multi-parameter flow cytometry allows simultaneous determination of several biochemical and physical variables immediately after sample preparation and hence should provide more accurate information on the cells’ physiological status than can be obtained by plating [38, 39]. This technique also offers key information on the heterogeneity of bacterial populations. Unlike plating, it does not underestimate the number of viable cells in original cultures in cases when a fraction of the population has experienced sublethal injury and lost the capacity to grow [39, 40].

Incubation of the three degraders and host controls was conducted under the conditions mentioned in the previous section. The samples withdrawn after 5 h incubation with or without TCP were stained with propidium iodide (PI), 6-carboxy-2′,7′-dichlorodihydrofluorescein diacetate (carboxy-H_2_DCFDA), or *bis*-(1,3-dibutylbarbituric acid) trimethine oxonol [DiBAC_4_(3)]. Dyes for labelling of nucleic acids, such as PI, which only enters cells with compromised membranes, are commonly used with membrane potential-sensitive dyes such as DiBAC_4_(3), which binds the lipid-containing intracellular components, to study bacterial viability [38, 39, 41]. Carboxy-H_2_DCFDA is a chemically reduced, acetylated and carboxylated form of fluorescein that has found many applications as a general indicator for the presence of reactive oxygen species (ROS) in eukaryotic and prokaryotic cells [42]. Recent studies on bacterial utilization of chlorinated aliphatic hydrocarbons suggest that this process is associated with strong oxidative stress and we hypothesized that TCP would also evoke such a physiological response [42, 43]. The saturation of unsaturated fatty acids by halogenation or lipid peroxidation causes changes in membrane fluidity, resulting in the collapse of electron transport chains and premature electron transfer to oxygen, accompanied by ROS formation, membrane permeabilization, and cell death [44, 45].

**Fig. 3:**
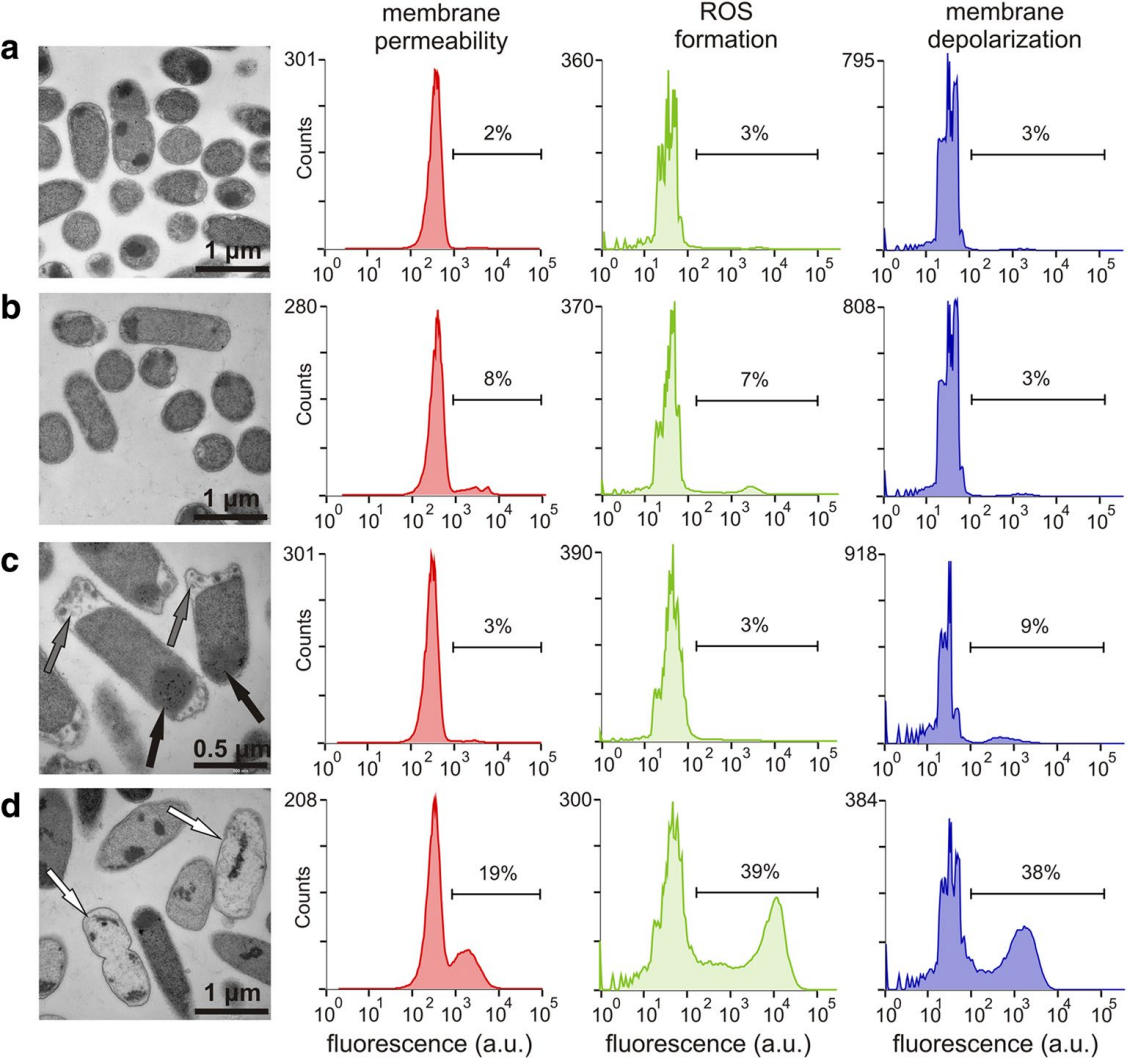
Transmission electron microscopy of *Escherichia coli* deg31 cells and corresponding *histograms* showing the physiological state of populations stained with selected fluorescent dyes. **a** Non-induced cells incubated in phosphate buffer. **b** Non-induced cells incubated in phosphate buffer with 2 mM TCP. **c** Cells pre-induced with 0.2 mM IPTG incubated in phosphate buffer. **d** Cells pre-induced with 0.2 mM IPTG and incubated in phosphate buffer with 2 mM TCP. *Black arrows* indicate bodies that presumably consist of overexpressed heterologous proteins, *grey arrows* indicate separations of the inner and outer cell membranes, and *white arrows* indicate dead or dying cells

The end-point flow cytometry protocol adopted in our study was less sensitive than plating in demonstrating the burden caused by plasmids (Fig. 2b), possibly because the presence of heterologous DNA and the constitutive expression of selection markers did not directly alter properties targeted by cytometry but instead imbalanced the cells’ overall energy status. However, the flow cytometry approach using selected fluorochromes was useful in exposing the toxic effects of IPTG and TCP. There were no significant differences (*P* > 0.05) between the non-induced host controls with empty plasmids, regardless of their TCP exposure status, with respect to any of the three variables examined in these experiments (membrane permeability, ROS formation, and membrane potential; see Fig. 2b). However, the proportion of cells staining positive for DiBAC_4_(3) increased up to threefold in the control treated with IPTG alone (*P* < 0.01). The same effect was also observed in deg31, whose response to induction and incubation with TCP was studied in more detail (Fig. 3). The fraction of the bacterial population staining positively with all three dyes increased many-fold when pre-treatment with IPTG was combined with TCP exposure, confirming the previously observed exacerbating effect and indicating that the action of TCP in bacterial cells is accompanied by extensive ROS formation (Figs. 2b, 3). Membrane depolarization, ROS accumulation, and membrane permeability were reduced in *E. coli* recombinants expressing the synthetic biodegradation pathway, with the degree of reduction being proportional to the strains’ initial rates of TCP conversion. Interestingly, the exacerbation of compound toxicity by pre-induction of the BL21(DE3) host control with IPTG was confirmed also in experiments using the model toxic compound *tert*-butyl hydroperoxide (TBHP), an organic peroxide and strong ROS formation promoting oxidative agent (Additional file 1: Fig. S3). This suggests that described exacerbation phenomenon should not be restricted only to our model toxic chemical TCP.

Membrane depolarization and ROS formation in vivo are dynamic processes. To follow their kinetics in *E. coli* degraders we also performed time-resolved measurements (Additional file 1: Fig. S4). The number of cells stained by DiBAC_4_(3) and carboxy-H_2_DCFDA increased linearly over time in all of the stressed recombinants except for deg31. Interestingly, this degrader exhibited an initial burst of DiBAC_4_(3) and carboxy-H_2_DCFDA fluorescence but these signals then plateaued or fell slightly. We assume that the characteristic profiles of DiBAC_4_(3) and carboxy-H_2_DCFDA fluorescence for deg31 are linked to its unique variant of the synthetic biodegradation pathway and the corresponding time-course of TCP biotransformation. In contrast to the other strains, TCP, 2,3-dichloropropane-1-ol (DCP) and glycidol (GDL) were present at relatively high concentrations in the deg31 reaction mixture between minutes 50 and 100 of the measurement period. This may have caused synergistic toxicity, increasing the number of cells with depolarized membranes and enhanced ROS formation. Such joint effects are common; they have been observed for organophosphate pesticides, fluorosurfactants, and heavy metals, among others [46–48]. Subsequent freezing or moderate fall in the intensity of the signals was attributed to the parallel removal of TCP and GDL and the production of glycerol, which is known to be an efficient stress protectant in yeast and solvent-tolerant strains of *E. coli* [49, 50].

The variant of the synthetic pathway present in deg31 seemed to provide the best compromise in terms of coping with toxicity while efficiently converting TCP into harmless glycerol and was therefore selected for further investigation.

### Assesment of metabolic burden and substrate toxicity effects by electron microscopy

Metabolic burden and toxicity can cause changes in the morphology of bacterial hosts [11, 51]. We therefore used transmission electron microscopy to study the changes in morphology of induced and non-induced deg31 cells after 5 h incubation with or without 2 mM TCP (Fig. 3). Pictures of induced and non-induced *E. coli* host control cells with empty plasmids are shown in (Additional file 1: Fig. S5). Microscopic observations were followed by multi-parameter flow cytometry of deg31 cells stained with PI, carboxy-H_2_DCFDA, or DiBAC_4_(3).

Incubation of non-induced degraders with TCP produced only a small proportion of dead bacterial cells, and the morphology of cells treated in this way did not generally differ from that of cells unexposed to the toxic substrate (Fig. 3a, b). The proportion of cells stained by carboxy-H_2_DCFDA and PI increased moderately, but no effect on the membrane potential was observed. Pre-induced bacteria producing recombinant proteins intensively formed visible inclusion bodies and showed frequent separation of the cytoplasmic membrane from the outer membrane at the poles of the cell (Fig. 3c). Since the majority of the recombinant proteins obtained from cell lysates were soluble (data not shown), we believe that the observed bodies consisted of active enzymes.

The combination of pre-induction and incubation with TCP produced the most pronounced morphological changes and was accompanied by substantial increases in the number of cells staining positively for PI, carboxy-H_2_DCFDA, and DiBAC_4_(3) (Fig. 3d). Numerous dead or dying cells with damaged cytoplasmic membranes and leaked contents were clearly visible. Even so, a significant portion of the population resisted the combined effects of IPTG and TCP over the 5 h treatment period. This was mainly due to the well-balanced synthetic pathway of deg31 and its fast conversion of TCP to glycerol. Bistability is a common phenomenon and can be attributed to noise in the expression of the multigenic traits responsible for toxicity tolerance and the graduated stress response in the bacterial population [29, 36, 52]. In summary, our microscopic observations of deg31 populations treated under four different conditions were consistent with previous results and supported the conclusion that pre-induction with IPTG exacerbates TCP toxicity.

### Toxicity exacerbation effect rises in cells experiencing metabolic burden from plasmids

Given that both the *E*. *coli* degraders and the host controls used in this work had to cope with the metabolic burden of the Duet plasmids and LacI^Q^/*P*_*lacUV5*_-T7 expression system, we decided to include *E. coli* controls without these components in the following experiment. For this purpose, we used *E. coli* BL21(DE3) without plasmids and the cloning strain *E. coli* DH5α, which lacks both the LacI^Q^/*P*_*lacUV5*_-T7 expression system and the *lac* operon. By employing the plating and flow cytometry protocols described above, we found that the exacerbation effect was modest or completely absent in both strains (Additional file 1: Fig. S6A, B). This suggests that the double stress from IPTG and TCP is only manifested in strains carrying Duet plasmids and the corresponding expression system.

We presume that the studied recombinants could not efficiently suppress the double stress from IPTG and the toxic substrate due to the metabolic burden imposed by plasmids and the corresponding shortage of resources required for cell maintenance. It seems that the chemical structure of IPTG, its transport or presence inside the cell triggers physiological changes that facilitate the manifestation of TCP toxicity in *E. coli* BL21(DE3) cells with Duet plasmids.

### Reducing metabolic burden and toxicity exacerbation by tuning IPTG concentration

**Fig. 4:**
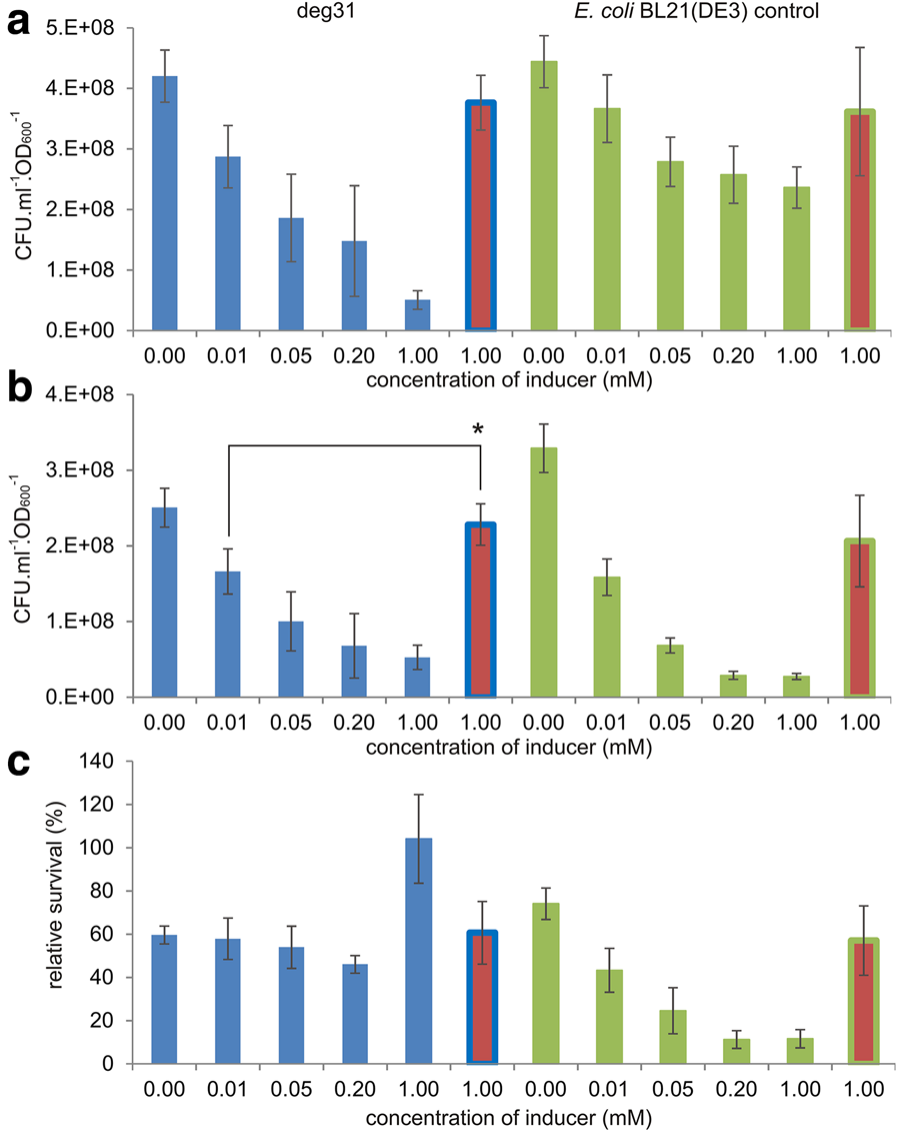
Viability of *Escherichia coli* deg31 and host control strains after pre-induction with diverse concentrations of IPTG or 1 mM lactose, before and after incubation with TCP. **a** Viability of deg31 and *E. coli* BL21(DE3) with the empty pETDuet and pCDF plasmids as determined by plating of cells pre-induced with different concentrations of IPTG or 1 mM lactose (*red columns*) before incubation in phosphate buffer with TCP. **b** Viability of cells after incubation in buffer with 2 mM TCP. *Asterisks* denote significantly higher (at *P* < 0.05) cell count of deg31 pre-induced with 1 mM lactose when compared with the count of cells pre-induced with the lowest tested concentration of IPTG (0.01 mM). **c** Fraction of surviving cells calculated as the difference in the CFUs.ml^−1^.OD_600_^−1^ before and after incubation with TCP. *Error bars* represent standard deviations calculated from at least four independent experiments. *CFU* colony forming units

Next, we attempted to reduce the burden imposed on *E. coli* recombinants by optimizing the concentration of IPTG. We looked for the lowest possible concentration of inducer that would minimize the fitness cost without significantly compromising the system’s efficiency at degrading TCP. Deg31 cells were pre-induced with IPTG concentrations ranging from 0.01 to 1.00 mM and the resulting effects on cell viability and pathway efficiency were studied (Fig. 4; Additional file 1: Fig. S7).

*E. coli* BL21(DE3) with the empty pETDuet and pCDF plasmids was used as a control for plating to assess the burden imposed on the host by IPTG exposure in the absence of the heterologous pathway (Fig. 4). The viability of the pre-induced degrader and control was checked before and after 5 h of incubation in phosphate buffer with 2 mM TCP and compared to that of cells not exposed to IPTG (Fig. 4a, b). The percentage of surviving cells after incubation was calculated in each case (Fig. 4c). These experiments indicated that even in the absence of TCP there was an inverse correlation between the IPTG concentration and the viability of the recombinant *E. coli* (Fig. 4a). The deg31 strain suffered more from increasing IPTG concentrations than the control, probably due to the additional burden of expressing genes encoding the synthetic pathway. The opposite was true after 5 h of incubation because the TCP-catabolizing deg31 strain was more resistant to exacerbated toxicity than the host control (Fig. 4b, c). The synthetic pathway strain exhibited similar survival rates at all tested IPTG concentrations except for the highest—the relative viability of deg31 pre-induced with 1 mM IPTG was 100 %. We assumed that this outlying value was due to underestimation of the number of viable cells before incubation due to the intensive stress experienced by the degrader upon induction with such a high concentration of IPTG and the resulting presence of viable-but-not-culturable cells in the suspension [40]. Plating experiments demonstrated that a fraction of the bacterial population regained its capacity to grow and reproduce some time after treatment with this high concentration of IPTG (Additional file 1: Fig. S8).

The time-courses of TCP biotransformation with resting cells of deg31 pre-induced with IPTG at 1.00, 0.20, 0.05, 0.025, and 0.01 mM (Additional file 1: Fig. S7) and densitometric analysis of SDS polyacrylamide gels with the corresponding samples of cell-free extracts (Additional file 1: Fig. S9, Table S1) showed that: (i) the relative ratio of pathway enzymes and the shape of the degradation profile did not change substantially with the inducer concentration, while (ii) the content of the three pathway enzymes in the total soluble protein of the recombinant bacteria decreased from 55 % (1 mM IPTG) to 32 % (0.01 mM IPTG) and the pathway’s output decreased from 70 to 46 %. Appreciable conversion of TCP was also achieved with non-induced cells due to the leakiness of the T7 promoter and basal expression of genes within the pathway (Additional file 1: Fig. S7).

**Fig. 5:**
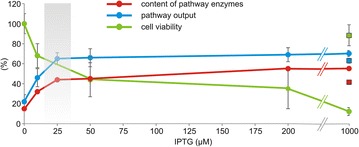
Summarized effects of IPTG concentration on gene expression levels, pathway output, and cell viability in pre-induced *Escherichia coli* deg31 cells. Viability was determined by plating pre-induced deg31 cells resuspended in phosphate buffer before incubation with TCP. Pathway output was expressed as the theoretical conversion of TCP into glycerol at the end of 5 h degradation experiments with pre-induced, resting deg31 cells (see also Additional file 1: Fig. S5). The content of TCP pathway enzymes was estimated by analyzing cell-free extracts obtained from pre-induced cells by sodium dodecyl sulfate polyacrylamide gel electrophoresis (Additional file 1: Fig. S7 and Table S1). Two gels were analysed by densitometry and mean values are shown. *Error bars* represent standard deviations calculated from three independent experiments. Values determined for deg31 pre-induced with 1 mM lactose are indicated by *squares*

Figure 5 summarizes the balance of the three parameters discussed in the preceding sections: (i) host viability, (ii) the cellular expression of pathway enzymes, and (iii) the output of the synthetic biodegradation route. We conclude that the minimal IPTG concentration that allows sufficient expression of genes within the pathway to achieve a reasonable output is 0.025 mM. Similar inducer concentrations that allow full gene expression have been reported for single recombinant proteins such as β-galactosidase, green fluorescent protein, and rhamnulose-1-phosphate aldolase [53–55]. Note that IPTG concentration of 0.025 mM is eight times lower than the originally tested inducer concentration and up to 40-times lower than the values reported in the scientific literature describing engineering of heterologous pathways in *E. coli* [9, 56]. Induction with lower amounts of IPTG improved the host’s fitness. However, even the lowest concentration of 0.01 mM reduced the viability of the *E. coli* degrader by 30 % relative to non-induced deg31, and by up to 50 % relative to the non-induced host control (*P* < 0.05 in both cases; Fig. 4b). We therefore investigated the scope for replacing IPTG with an alternative inducer.

### Reducing metabolic burden and toxicity exacerbation by inducing with lactose

Lactose is a natural inducer of the *lac* operon and can be employed as a cheaper alternative to synthetic IPTG. It has been proven to induce expression of recombinant proteins in *E. coli* to the same extent as IPTG on both laboratory and industrial scales [12, 57, 58]. In contrast to IPTG, lactose is a substrate of β-galactosidase (encoded by *lacZ*) and thus can be metabolized by cells with an intact *lac* operon, including *E. coli* BL21(DE3). Therefore, concentrations of lactose up to 30 mM are commonly used to induce the expression of cloned genes at levels that can be achieved with ≤1 mM IPTG [58].

We investigated the pre-induction of deg31 cells with 1 mM lactose. We assumed that this concentration would be sufficient to induce the expression of the TCP degradation pathway genes at levels that would confer adequate degradation efficiency. This expectation was confirmed by recorded time-courses of TCP biotransformation and the finding that pathway enzymes accounted for up to 41 % of the cells’ total soluble protein under these conditions (Additional file 1: Figs. S7 and S9, and Table S1). The theoretical conversion of TCP into glycerol under these conditions was 63 %. These values are close to those observed for deg31 cells pre-induced with 0.025 or 0.05 mM IPTG. Most importantly, the deg31 cells pre-induced with lactose exhibited higher viability before and after 5 h incubation with TCP compared to bacteria treated with IPTG at any of the concentrations tested (P < 0.05; Fig. 4). The same relieving effect was observed for the host control. In term of survival, the cells pre-induced with lactose performed almost as well as their non-induced counterparts (Fig. 4c). In keeping with the plating results, flow cytometric analysis of host control and deg31 cells pre-induced with lactose revealed significantly lower levels of stressed cells with depolarized membranes than were observed for *E. coli* strains pre-induced with IPTG (P < 0.01; Additional file 1: Fig. S10). These results indicated again that the action of IPTG in the studied recombinants was consistently accompanied by changes in membrane properties.

A higher viability of cells induced with lactose instead of IPTG was previously reported for the expression of single heterologous proteins [12, 56, 58]. This effect was attributed to the delayed, milder induction achieved with the natural inducer. In contrast to synthetic IPTG, which can enter the cell rapidly both by passive diffusion and with the help of the LacY lactose permease, lactose can only enter the cytoplasm via the permease. Moreover, lactose must be converted into allolactose by β-galactosidase before binding to the *lac* repressor whereas IPTG binds to the repressor directly. Our results confirm that lactose imposes a lower metabolic burden than IPTG, suggesting that it is also a more suitable inducer for the expression of entire heterologous pathways in *E. coli* BL21(DE3). This is particularly important for *E. coli* BL21(DE3) cells carrying synthetic pathways that degrade toxic compounds or produce toxic intermediates.

## Conclusions

We studied the toxicity of TCP and its derived metabolites in *E. coli* BL21(DE3) carrying an inducible synthetic metabolic pathway comprising three catabolic genes [22]. The presence of plasmid DNA, exposure to IPTG, and the metabolic burden of the heterologous pathway were shown to affect the viability of *E. coli* degraders even before contact with the toxic substrate. The most pronounced impact was attributed to plasmid maintenance: the presence of two medium-to-high copy number plasmids (pCDF and pETDuet) reduced cell viability by 50 %. Pre-induction of host control cells carrying the empty plasmids caused a 40 % decrease in viability, while expression of the genes within the pathway further reduced viability by about 20 %. Treatment with exogenous TCP at a concentration of 2 mM had only a very minor or no effect on the viability of non-induced control cells carrying empty plasmids. The pronounced reduction in survival triggered by adding the substrate to pre-induced cells was unexpected, suggesting that TCP toxicity was exacerbated by pretreatment of the cells with IPTG. The recombinants clearly benefited from the presence of a functioning TCP degradation pathway—the faster the conversion of TCP, the greater the cell viability.

Flow cytometry with selected fluorochromes proved to be useful in dissecting the toxic effects of IPTG and TCP. Variations in the three monitored physiological parameters—membrane permeability, ROS formation, and membrane potential—was insignificant between the non-induced host controls irrespective of exposure to TCP. The fraction of cells staining positively for individual dyes rose significantly when IPTG was added together with TCP, verifying the previously observed exacerbation effect and showing that TCP exposure causes intensive ROS formation in bacterial cells. All three parameters were reduced in *E. coli* BL21(DE3) recombinants expressing the synthetic biodegradation pathway, and the degree of reduction increased in parallel with the initial rate of TCP degradation. Cell counts and flow cytometry data were in good agreement with the results of electron microscopy experiments.

The potential toxicity of IPTG and its negative effect on cell growth has been repeatedly reported [9, 16, 60, 61]. This effect was attributed to various factors including the induced changes in the host metabolism [16], the active uptake of the inducer into the cell by LacY [17, 62], and the rapid induction of heterologous gene expression [12, 56, 59]. However, that widely used inducer can exacerbate the toxicity of another compound, leading to damage of host cells experiencing metabolic burden, has not been previously reported and should be of wide interest. *E. coli* BL21(DE3) or similar hosts with the DE3 lysogen and well defined plasmid vectors are widely used because they make it possible to precisely balance the expression of heterologous pathway genes in metabolic engineering and synthetic biology [8, 10, 22, 25]. Still, insufficient attention has been paid to the possible exacerbation of multiple endogenous and exogenous stressors that impose a complex burden on such cell factories. IPTG is often applied at sub-millimolar concentrations [9, 63, 64] although the amount needed for full induction of heterologous genes can be an order of magnitude lower. The optimal concentration of IPTG in any given case may be system-specific [56], but the relatively simple experiments can be used to guide the optimization of its concentration. Importantly, the use of lactose as an alternative inducer should be considered: we observed that replacing IPTG with lactose dramatically reduced the burden experienced by the transformed bacterial cells, suggesting that it may be a better inducer than IPTG for the expression of heterologous pathways in *E. coli* BL21(DE3).

## Methods

### Chemicals and growth media

TCP, DCP, epichlorohydrin (ECH), 3-chloropropane-1,2-diol (CPD), GDL, and glycerol standards were purchased from Sigma-Aldrich Co. (St. Louis, MO, USA). All chemicals used in this study were of analytical grade. The fluorescent indicators carboxy-H_2_DCFDA and DiBAC_4_(3) were purchased from Life Technologies Inc. (Waltham, MA, USA) and PI from Sigma-Aldrich Co. IPTG was purchased from Duchefa Biochemie B.V. (Haarlem, The Netherlands). Lactose and TBHP solution (5–6 M in decane) were from Sigma-Aldrich Co. A Free Glycerol Assay Kit was acquired from BioVision Inc. (Milpitas, CA, USA). LB medium (Sigma-Aldrich Co.) was used for routine cultivation of *E*. *coli*.

### Bacterial strains and growth conditions

Plasmids and *E. coli* strains used in this study are summarized in Table 1. *E. coli* DH5α was used as a control strain lacking *lacZYA* operon. *E. coli* BL21(DE3) was used as a heterologous host for inducible expression of the synthetic biodegradation pathway and also as a control strain bearing the *lacZYA* operon and LacI^Q^/*P*_*lacUV5*_-T7 expression system. Three variants of the pathway were constructed previously in a modular way by combining genes encoding haloalkane dehalogenase DhaA from *R. rhodochrous* NCIMB 13064, the DhaA mutant DhaA31, and genes encoding haloalcohol dehalogenase HheC and epoxide hydrolase EchA from *A. radiobacter* AD1 on Duet vectors [22]. For the purpose of this study, *E. coli* BL21(DE3) degraders and host control strain BL21(DE3) with empty pCDF and pETDuet plasmids were prepared freshly by co-transforming plasmid constructs into competent cells using chemical transformation or electroporation. Cells were spread on LB agar plates with appropriate combination of antibiotics (25 μg.ml^−1^ Sm and 75 μg.ml^−1^ Amp for *E*. *coli* BL21(DE3) with empty pCDF and pETDuet, degWT, degWTopt, and deg31; 25 μg.ml^−1^ Sm and 20 μg.ml^−1^ Cm for deg31opt) and grown overnight at 37 °C. A single colony was used for preparation of overnight cultures in 10 ml of LB medium with the corresponding antibiotics. Precultures (250 µl) were used to inoculate fresh LB medium (25 ml) and cultures were grown at 37 °C with shaking until the cell density reached an optical density measured at 600 nm (OD_600_) of 1 when the expression of recombinant genes was induced with IPTG or lactose. Induced cells were incubated overnight at 20 °C and biomass was collected at late exponential phase by centrifugation at 4000*g* for 10 min (4 °C). Pellets were washed with ice-cold sterile filtered sodium phosphate buffer (50 mM, pH 7.0) and dissolved in the same buffer for further use.

### Incubation of resting cells with TCP and assessment of viability by plating

Cell suspensions of pre-induced or non-induced *E. coli* degraders and host cell controls were diluted with sterile filtered sodium phosphate buffer to a final OD_600_ of 7.0. TCP (4 mM) was dissolved in 5 ml of the same buffer in glass vials (25 ml) with a screw cap mininert valve for 1 h at 37 °C with shaking. The incubation was initiated by mixing 5 ml of the cell suspension with 5 ml of buffer with TCP. The final TCP concentration in 10 ml of the cell suspension of OD_600_ of 3.5 was 2 mM. In the cases indicated, 1 mM TBHP was added instead of TCP. In case of *E. coli* degraders, cell suspension samples (0.5 ml) were quenched in 0.5 ml acetone with hexan-1-ol, vortexed for 15 s and centrifuged at 18,000*g* for 2 min. The concentration of metabolites from the TCP pathway in the supernatant was analyzed using gas chromatography. The presence of GLY in the cell suspension was verified using a Free Glycerol Assay Kit (BioVision Inc.). Viability of degraders and controls before and after 2.5 or 5 h incubation with TCP was tested by plating 100 µl of cell suspension serially diluted with ice-cold sterile phosphate-buffered saline (PBS, 8 mM Na_2_HPO_4_, 1.5 mM KH_2_PO_4_, 3 mM KCl, and 137 mM NaCl, pH = 7.0) onto Plate Count Agar plates. Plates were incubated for 24 h at 37 °C and grown colonies were counted as CFU.ml^−1^.OD_600_^−1^.

### End-point flow cytometry

At the end of incubation of *E. coli* degraders and host cell controls with or without TCP, 0.1 ml of each cell suspension was added to 0.9 ml of filtered PBS. The suspensions were added with carboxy-H_2_DCFDA (prepared as 4 mM stock solution in DMSO) to the final concentration of 40 μM and with PI (prepared as 200 μg.ml^−1^ stock solution in PBS) to the final concentration of 2 μg.ml^−1^ or with 1 μM DiBAC_4_(3) (prepared as 25 μg.ml^−1^ stock solution in 4 mM EDTA). The suspensions with fluorescent probes were mixed by inverting and incubated in the dark for 10 min at room temperature. Flow cytometry analysis of fluorescence levels was performed using BD FACSAria II Sorp (BD Biosciences Co., San José, CA, USA) equipped with an argon-ion laser of 100 mW at 488 nm and a solid state 100 mW 561 nm as the excitation source. The carboxy-H_2_DCFDA or DiBAC_4_(3) fluorescence emission at 525 or 516 nm, respectively, was detected using a 525/50-nm band pass filter array. The PI fluorescence emission at 617 nm was detected using a 610/20-nm band pass filter array. Data for at least 50,000 cells per single sample per experiment were collected. Size-related forward scatter signals gathered by the cytometer were used by the Cyflogic 1.2.1 software (CyFlo Ltd., Finland) to gate fluorescence data from bacteria in the stream. Percentage of cells positive for carboxy-H_2_DCFDA, PI and DiBAC_4_(3) was calculated using the statistics module of the software.

### Time-resolved flow cytometry

For time-resolved measurements of ROS accumulation in *E. coli* constructs, 0.5 ml of the cell suspension in sterile filtered sodium phosphate buffer (50 mM, pH 7.0) of OD_600_ of 7.0 was added with carboxy-H_2_DCFDA at 40 μM. The suspension was mixed by inverting and incubated in the dark for 10 min at room temperature. The measurement was started immediately after mixing the cell suspension with 0.5 ml of sodium phosphate buffer with dissolved TCP in 5 ml polypropylene tube with snap cap. The final TCP concentration in 1 ml of cell suspension of OD_600_ of 3.5 was 2 mM. Flow cytometry analysis of fluorescence levels in time was performed on the same FACS instrumentation as above. FCS data were recorded in BD FACS Diva (v. 6.1.3, BD Biosciences Co.) at constant flow rate of 20,000 events.s^−1^ for intervals of 5 s every 30 min for total period of 5 h. Analysis of data was further performed in FlowJo LLC. (v. 7.6.5, Ashland, OR, USA) and exported to Excel (Office 2013, Microsoft Corp., Redmond, WA, USA) for graphical output. Time-resolved profiles of membrane depolarization in *E. coli* constructs during 5 h incubation in phosphate buffer were compiled from end-point measurements performed at time 0, 1, 2, 3, 4, and 5 h following the procedure described in previous section.

### Determination of expression levels of the TCP pathway enzymes

The expression levels of DhaA31, HheC, and EchA were determined in *E. coli* constructs induced with diverse concentrations of IPTG or 1 mM lactose. Washed cells were resuspended in sodium phosphate buffer and cell density was adjusted to OD_600_ of 7.0. 1 U of DNaseI per 1 ml of cell suspension was added. Cells were disrupted with One Shot cell disruptor (Constant Systems Ltd., UK) using 1.5 kbar shot. The cell lysate was centrifuged for 45 min at 18,000*g* at 4 °C and the resulting cell-free extract (CFE) was decanted. The concentration of total protein in CFEs was determined using Bradford reagent (Sigma Aldrich Co., USA). Samples of CFE containing 5 μg of total protein were separated by sodium dodecyl sulfate polyacrylamide gel electrophoresis (SDS-PAGE). CFE prepared from *E. coli* BL21 (DE3) cells without plasmids were used as controls. Gels were stained with Coomassie Brilliant Blue R-250 (Fluka/Sigma-Aldrich Co., Switzerland) and analyzed using a GS-800 Calibrated Imaging Densitometer (Bio-Rad Laboraories Inc., USA). The expression levels of DhaA31, HheC and EchA and the relative ratios of the enzymes in degWT, deg31, and deg31opt were estimated from trace densities of corresponding bands using the software Quantity One 4.6.9 (Bio-Rad Laboratories Inc., USA).

### Electron microscopy

To observe morphological changes, cells of *E. coli* deg31 induced with 0.2 mM IPTG or non-induced were incubated in presence or absence of 2 mM TCP in sodium phosphate buffer (50 mM, pH 7.0, OD_600_ of cell suspension of 3.5) at 37 °C for 5 h and then processed for transmission electron microscopy. Briefly, cells were harvested and pelleted by centrifugation (5000*g*, 5 min). Pellets were washed and resuspended in phosphate buffer and fixed in 3 % glutaraldehyde solution in the same buffer for 1 h at room temperature. Cells were then post-fixed in 1 % osmium tetroxide in phosphate buffer and after addition of 2.5 % agar and dehydration in a graded series of ethanol, the blocks of cells were impregnated with Durcupan (Sigma-Aldrich Co., USA) and embedded in silicone embedding moulds. Polymerization occurred for 3 days at 60–80 °C. Ultrathin sections prepared with a diamond knife on an Ultramicrotome EM UC6 (Leica Microsystems GmbH, Germany) were placed on copper grids, stained with 2.5 % uranyl acetate for 10 min and Reynolds lead citrate solution for 3 min and observed with a Morgagni 268D (FEI Co., The Netherlands) transmission electron microscope.

### Gas chromatography and mass spectrometry measurements

A Gas Chromatograph 6890 N with a flame ionisation detector (GC-FID) and Gas Chromatograph 7890A and Mass Spectrometer (GC–MS) 5975C MSD (Agilent Technologies Inc., USA), both with the capillary column ZB-FFAP 30 m × 0.25 mm × 0.25 µm (Phenomenex Inc., USA) were used for routine analysis and quantification of TCP and its metabolites or for verification of the presence of individual metabolites from the TCP pathway in selected samples, respectively. Samples (2 μl) were injected into the GC with an inlet temperature of 250 °C and split ratio 20:1. The operational conditions for the column were: helium carrier gas with an initial flow of 0.6 ml.min^−1^ for 1 min, followed by a flow gradient from 0.6 to 1.8 ml.min^−1^ (ramp 0.2 ml.min^−1^), temperature program set to give an initial column temperature of 50 °C for 1 min, followed by a temperature gradient from 50 to 220 °C hold for 2 min (ramp 25 °C.min^−1^). The temperature of the detector was 250 °C. MS scan speed was 6.9 s^−1^. This method was used for all GC analyses. For that purpose, calibration curve of 0–5 mM of TCP, DCP, ECH, CPD and GDL with internal standard hexan-1-ol was prepared. Detection limits calculated using the software OriginPro v8 (OriginLab Corporation, USA) were 3, 5, 6, 186 and 22 μM for TCP, DCP, ECH, CPD and GDL, respectively.

### Statistical analysis

All experiments were independently repeated at least three times (number of repetitions is specified in figure legends) and the mean value of the corresponding parameter ± standard deviation is presented. Statistical significance was assessed using Student’s *t* test with two-tailed hypothesis available in Microsoft Excel 2013 (Microsoft Corp., USA). The difference in between two independent data sets was considered statistically significant for *P* < 0.05.

## Additional file


10.1186/s12934-015-0393-3 Supplementary material.

## Abbreviations

IPTG: isopropyl β-D-1-thiogalactopyranoside
TCP: 1,2,3-trichloropropane
PI: propidium iodide
carboxy-H_2_DCFDA: 6-carboxy-2′,7′-dichlorodihydrofluorescein diacetate
DiBAC_4_(3): *bis*-(1,3-dibutylbarbituric acid)trimethine oxonol
TBHP: *tert*-butyl hydroperoxide
