# Supplementary material for: Exacerbation of substrate toxicity by IPTG in Escherichia coli BL21(DE3) carrying a synthetic metabolic pathway
Source: Microb Cell Fact. 2015 Dec 21;14:201. doi: 10.1186/s12934-015-0393-3 (PMC4687329; doi:10.1186/s12934-015-0393-3)
Supplement: Supplementary file 1 — 10.1186/s12934-015-0393-3 Supplementary material. [file 12934_2015_393_MOESM1_ESM.docx]

**Additional File 1**

**
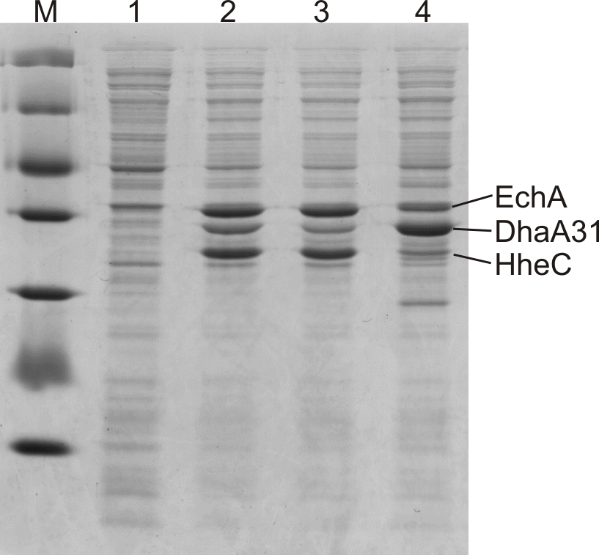
**

**Figure S1. Sodium dodecyl sulfate polyacrylamide gel electrophoresis of cell free extracts obtained from *Escherichia coli* degraders and host control induced with 0.2 mM IPTG.** M, protein marker (116, 66.2, 45, 35, 25, 18.4, 14.4 kDa); 1, host control with empty plasmids pETDuet and pCDF; 2, degWT; 3, deg31; 4, deg31opt. Bands of haloalkane dehalogenase mutant DhaA31 (34 kDa), haloalcohol dehalogenase HheC (29 kDa) and epoxide hydrolase EchA (35 kDa) are marked.

**
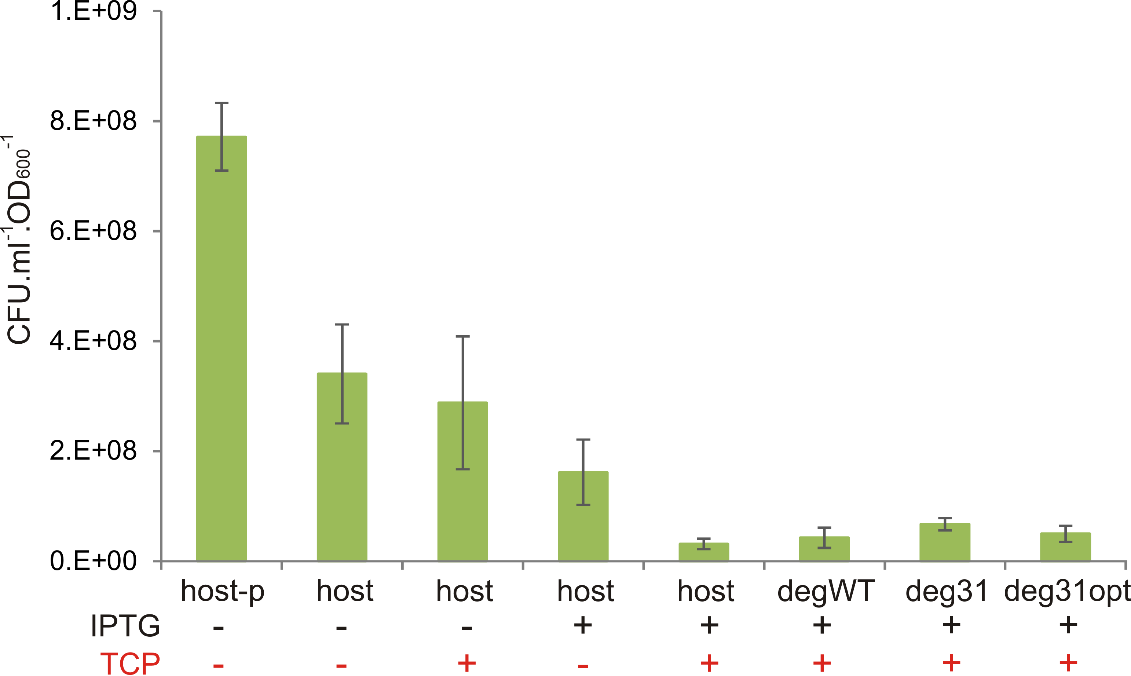
**

**Figure S2. Viability of *Escherichia coli* BL21(DE3) host controls and *E. coli* degraders determined by plating after incubation in phosphate buffer.** Error bars represent standard deviations calculated from at least five independent experiments. Abbreviations: CFU, colony forming units; host-p, *E. coli* BL21(DE3) without plasmids; host, *E. coli* BL21(DE3) with empty plasmids pETDuet and pCDF; +IPTG, pre-induction with 0.2 mM IPTG; +TCP, incubation with 2 mM TCP.


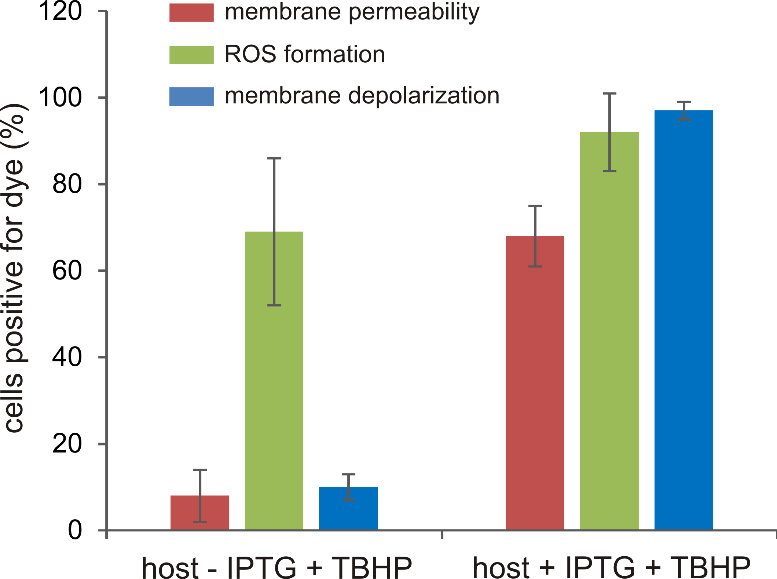


**Figure S3.** **Membrane permeability, oxidative stress and membrane depolarization in populations of *Escherichia coli* host control non-induced or pre-induced with 0.2 mM IPTG.** The parameters were determined by flow cytometry after incubation of cells in phosphate buffer with 1 mM *tert*-Butyl hydroperoxide and subsequent staining with respective fluorescent dyes. Error bars represent standard deviations calculated from four independent experiments. Abbreviation: host, *E. coli* BL21(DE3) with empty plasmids pETDuet and pCDF.

**
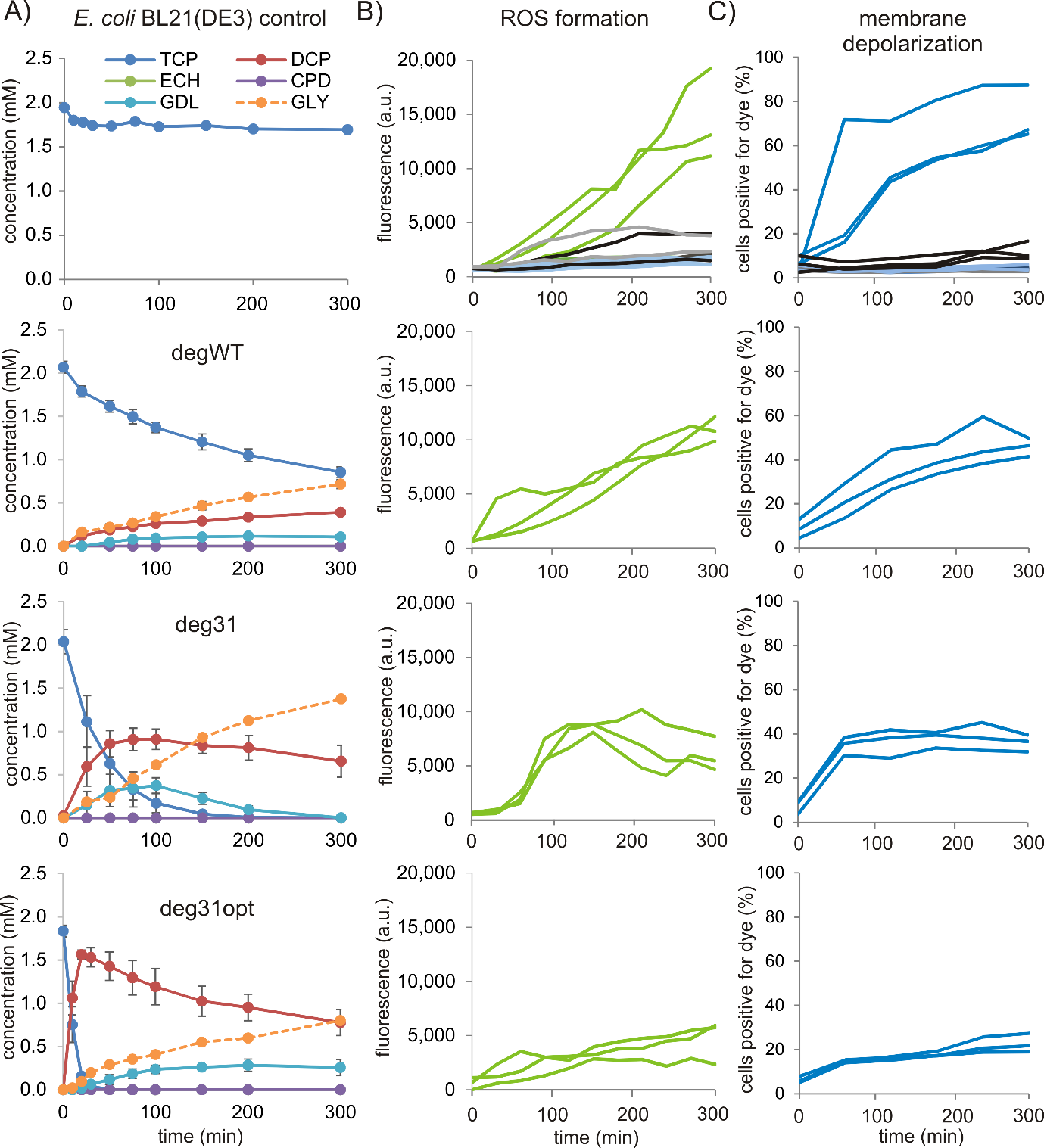
**

**Figure S4. TCP biotransformations catalyzed by *Esherichia coli* BL21(DE3) host control and constructs with synthetic pathway and corresponding time-resolved measurements of oxidative stress and membrane depolarization.** **A)** TCP biotransformations catalyzed by *Esherichia coli* BL21(DE3) with empty plasmids pETDuet+pCDF and three degraders. **B)** Corresponding time-resolved measurements of oxidative stress by flow cytometry after staining cells with carboxy-H_2_DCFDA. **C)** Corresponding time-resolved measurements of membrane depolarization by flow cytometry after staining with DiBAC_4_(3). Fluorescence was recorded every 30 min after addition of carboxy-H_2_DCFDA into the cell suspensions prior to the measurement. The same strategy applied for DiBAC_4_(3) did not provide reliable outcome. Therefore, standard end-point measurement of fluorescence conducted every 1 h after start of incubation was utilized. Data from three independent experiments are shown as coherent lines for sake of clarity. Black lines in top B) and C) graphs represent control of *E. coli* BL21 (DE3) cells bearing empty plasmids pETDuet and pCDF, pre-induced with 0.2 mM IPTG but not exposed to TCP. Grey lines represent non-induced control exposed to 2 mM TCP and pale blue lines represent non-induced control not exposed to TCP. Abbreviations: DCP, 2,3-dichloropropan-1-ol; ECH, epichlorohydrin; CPD, 3-chloropropane-1,2-diol; GDL, glycidol; GLY, glycerol.


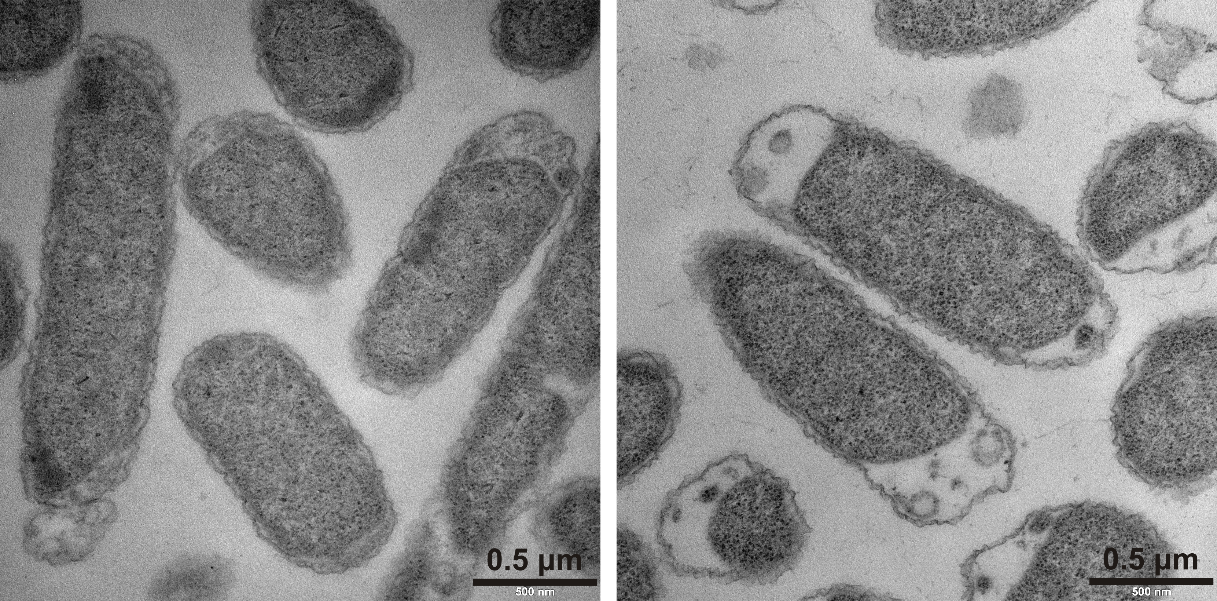


**Figure S5**. **Transmission electron microscopy of *Escherichia coli* host control carrying the empty pCDF and pETDuet plasmids.** Non-induced cells incubated in phosphate buffer (on left) and cells pre-induced with 0.2 mM IPTG and incubated in phosphate buffer (on right).


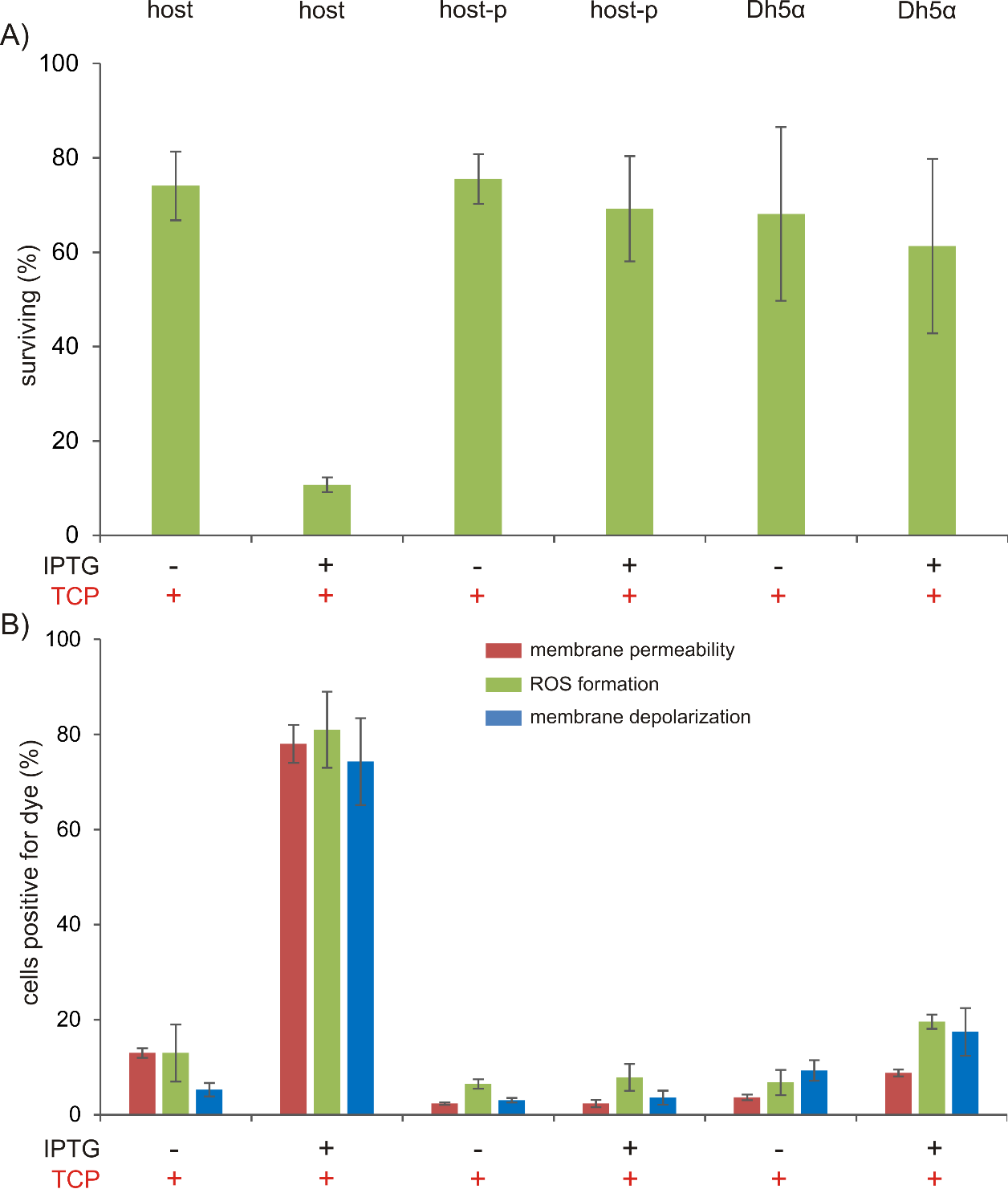


**Figure S6**. **The exacerbation of TCP toxicity by pre-treatment with 0.2 mM IPTG in three *Escherichia coli* control strains.** **A)** The percentage of surviving cells after incubation in phosphate buffer with 2 mM TCP. **B)** Membrane permeability, oxidative stress and membrane depolarization in populations of *E. coli* controls determined by flow cytometry after incubation in buffer and subsequent staining with respective fluorescent dyes. Error bars represent standard deviations calculated from five independent experiments. Abbreviations: host, *E. coli* BL21(DE3) with empty plasmids pETDuet+pCDF; host-p, *E. coli* BL21(DE3) without plasmids; DH5α, *E. coli* DH5α.


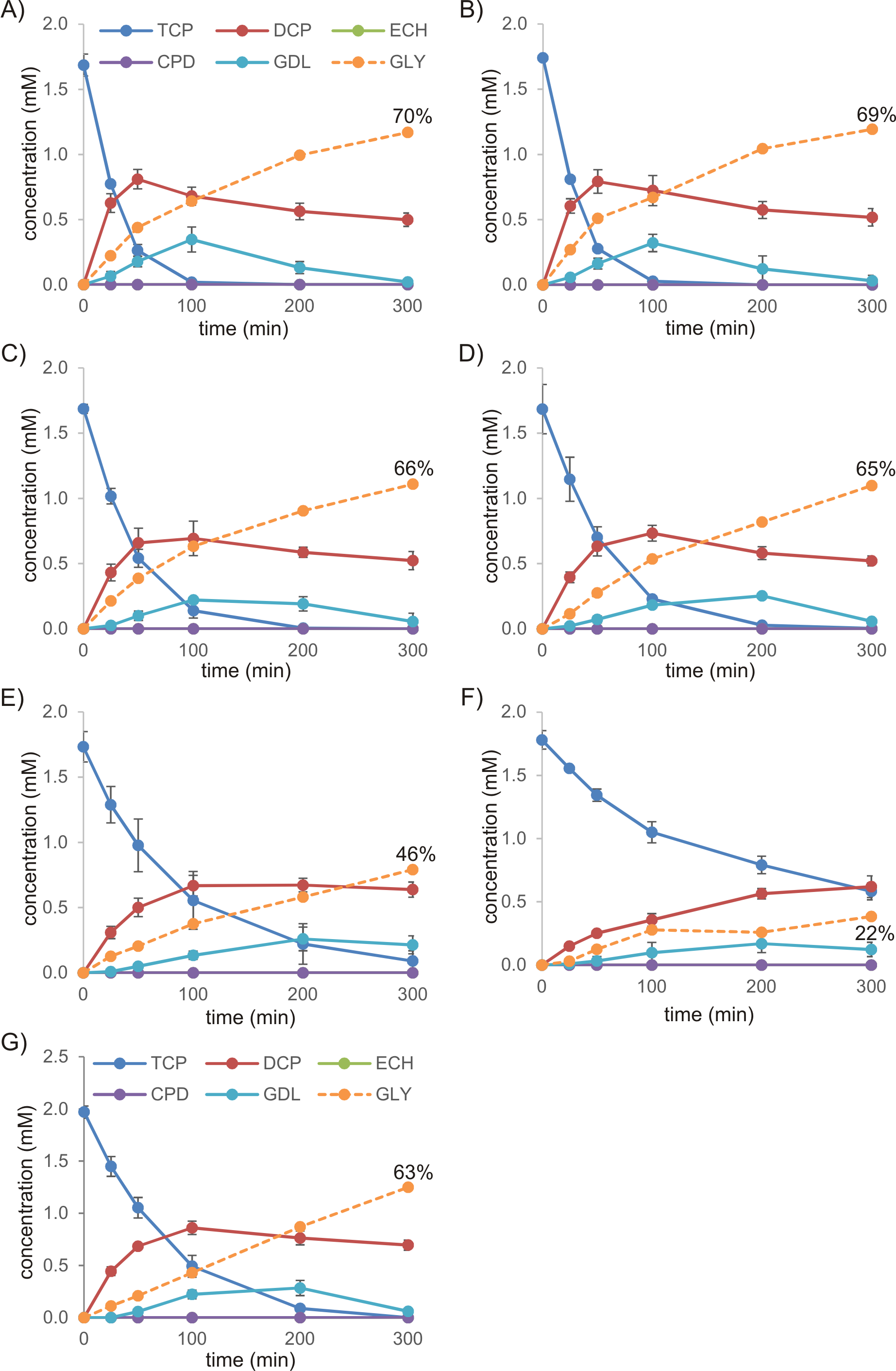


**Figure S7. TCP biotransformations catalyzed by *Esherichia coli* deg31 cells pre-induced with diverse concentration of IPTG or with 1 mM lactose.** **A)** 1.0 mM IPTG, **B)** 0.2 mM IPTG, **C)** 0.05 mM IPTG, **D)** 0.025 mM IPTG, **E)** 0.01 mM IPTG, **F)** 0 mM IPTG, **G)** 1 mM lactose. Theoretical conversions of TCP to glycerol (GLY; pathway output) are shown in %. Theoretical concentrations of GLY were calculated from concentrations of TCP and intermediates determined in experimental reactions. Abbreviations: DCP, 2,3-dichloropropan-1-ol; ECH, epichlorohydrin; CPD, 3-chloropropane-1,2-diol; GDL, glycidol.


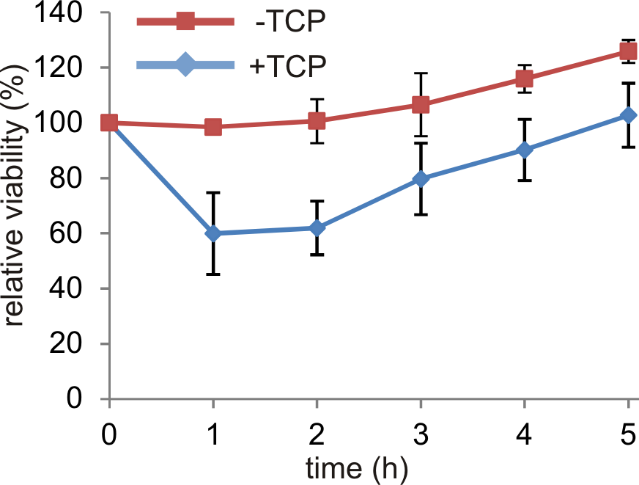


**Figure S8. Viability of deg31 cells pre-induced with 1 mM IPTG determined by plating during incubation in phosphate buffer with or without 2 mM TCP.**


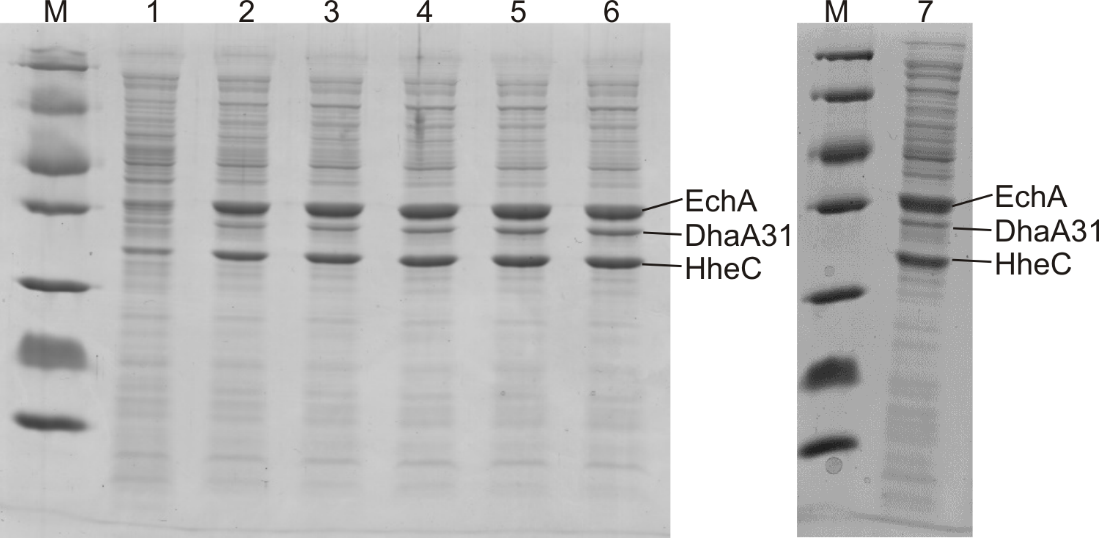


**Figure S9. Sodium dodecyl sulfate polyacrylamide gel electrophoresis of cell free extracts obtained from *Escherichia coli* deg31 cells induced with diverse concentrations of IPTG or 1 mM lactose.** M, protein marker (116, 66.2, 45, 35, 25, 18.4, 14.4 kDa); 1, 0 mM IPTG; 2, 0.01 mM IPTG; 3, 0.025 mM IPTG; 4, 0.05 mM IPTG; 5, 0.2 mM IPTG; 6, 1.0 mM IPTG; 7, 1 mM lactose. Bands of haloalkane dehalogenase mutant DhaA31 (34 kDa), haloalcohol dehalogenase HheC (29 kDa) and epoxide hydrolase EchA (35 kDa) are marked.

**Table S1. Content and relative ratio of enzymes from synthetic pathway for biotransformation of TCP determined in cell-free extracts of *Escherichia coli* deg31 cells induced with diverse concentrations of IPTG or 1 mM lactose.**

| IPTG (mM) | content in cell-free extract (%)* | relative ratio of enzymes (DhaA31:HheC:EchA) |
| --- | --- | --- |
| 0.00 | 15 | 0.18 : 0.37 : 0.45 |
| 0.01 | 32 | 0.13 : 0.37 : 0.50 |
| 0.025 | 45 | 0.12 : 0.38 : 0.50 |
| 0.05 | 45 | 0.13 : 0.38 : 0.49 |
| 0.20 | 55 | 0.16 : 0.39 : 0.45 |
| 1.00 | 55 | 0.17 : 0.39 : 0.44 |
| lactose (mM) |  |  |
| 1.00 | 41 | 0.12 : 0.42 : 0.46 |

*Calculated as a portion of total soluble protein content.


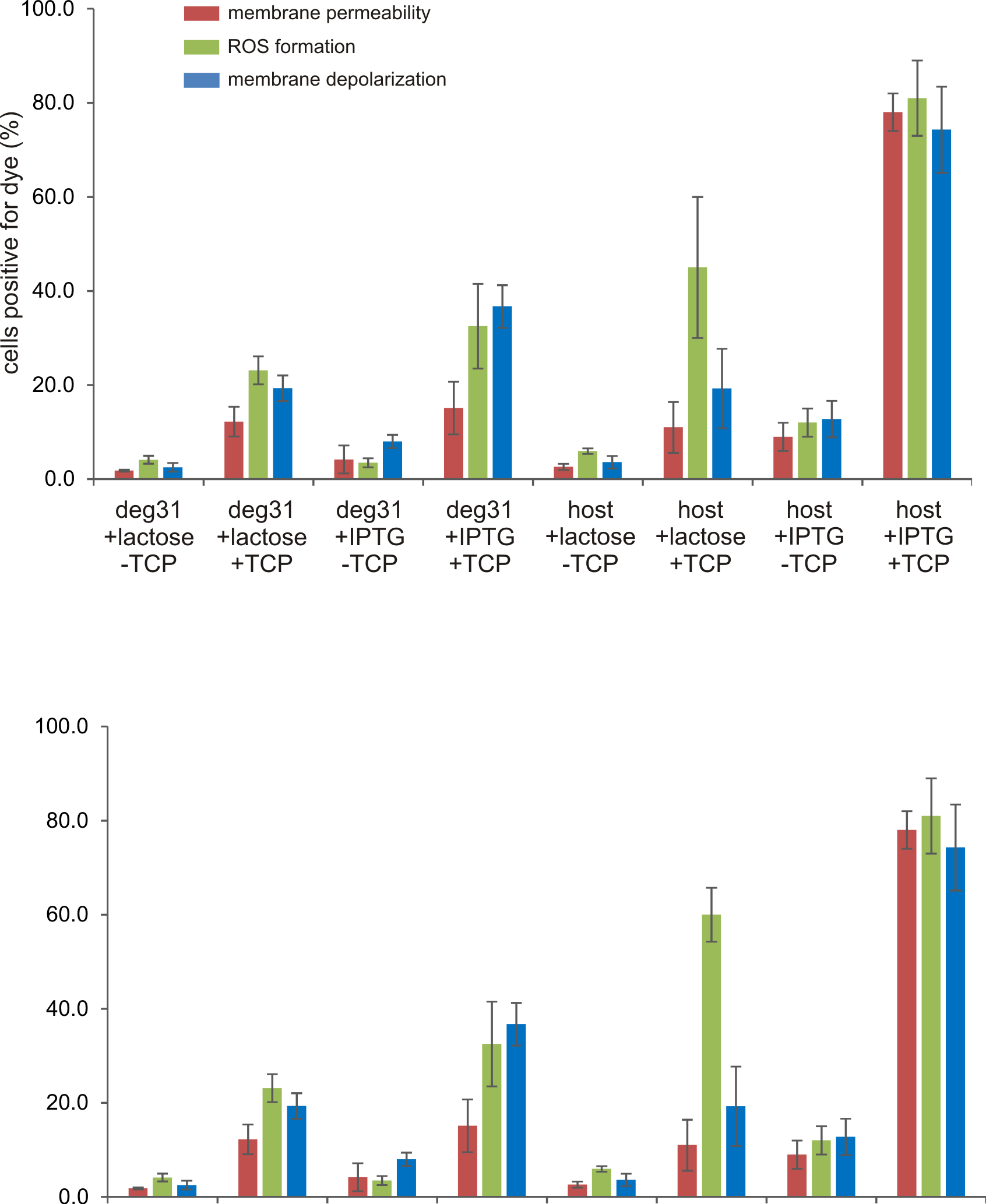


**Figure S10. Membrane permeability, oxidative stress and membrane depolarization in populations of deg31 cells and *Escherichia coli* host control pre-induced with 1 mM lactose or 0.2 mM IPTG.** The parameters were determined by flow cytometry after incubation of cells in phosphate buffer with or without 2 mM TCP and subsequent staining with respective fluorescent dyes. Error bars represent standard deviations calculated from four independent experiments. Abbreviation: host, *E. coli* BL21(DE3) with empty plasmids pETDuet+pCDF.
